# Health impacts of exposure to second hand smoke (SHS) amongst a highly exposed workforce: survey of London casino workers

**DOI:** 10.1186/1471-2458-7-257

**Published:** 2007-09-21

**Authors:** Paul A Pilkington, Selena Gray, Anna B Gilmore

**Affiliations:** 1 Centre for Public Health Research, University of the West of England, Bristol, UK; 2 European Centre on Health of Societies in Transition, London School of Hygiene and Tropical Medicine, London, UK

## Abstract

**Background:**

Casino workers are exposed to high levels of secondhand smoke (SHS) at work, yet remain at risk of being excluded from smoke-free legislation around the world. If the prime motivation for smoke-free legislation is the protection of workers, then a workforce experiencing ill-health associated with SHS exposure should not be excluded from legislation. This study aimed to determine the prevalence of respiratory and sensory irritation symptoms among a sample of casino workers, to identify any association between the reporting of symptoms and exposure to SHS at work, and to compare the prevalence of symptoms with that in other workers exposed to SHS.

**Methods:**

A postal questionnaire survey of 1568 casino workers in London. Using multivariate analysis we identified predictors of respiratory and sensory irritation symptoms.

**Results:**

559 workers responded to the questionnaire (response of 36%). 91% of casino workers reported the presence of one or more sensory irritation symptoms in the previous four weeks, while the figure was 84% for respiratory symptoms. The presence of one or more sensory irritation symptoms was most strongly associated with reporting the highest exposure to SHS at work (OR 3.26; 1.72, 6.16). This was also true for reporting the presence of one or more respiratory irritation symptoms (OR 2.24; 1.34, 3.74). Prevalence of irritation symptoms in the casino workers was in general appreciably higher than that reported in studies of bar workers.

**Conclusion:**

Our research supports the need for comprehensive smoke-free legislation around the world, covering all indoor workplaces including casinos.

## Background

In recent years, following conclusions from authoritative groups that second hand smoke (SHS) is harmful to health, policy makers around the world have increasingly sought to remove it from workplaces through smoke-free legislation [1-3]. The prime motivation for this policy move is the protection of workers from the negative health effects of SHS. However, those workers most exposed to SHS are often most at risk of remaining unprotected from smoke-free legislation. For instance, exemptions are often sought for the hospitality and gaming sectors, on the basis that trade will suffer because smokers will no longer visit these premises if they cannot smoke [4,5]. This is despite evidence that the introduction of smoke-free legislation does not lead to loss of business in the hospitality industry [6].

Research evidence suggests that casino workers, and those visiting casinos, are exposed to particularly high levels of SHS [7-10]. There are examples of smoke-free legislation that does include casinos, for example in Ireland, Norway and Italy [11-13]. However, casino workers risk being, or are already, excluded from smoke-free legislation in other parts of the world. For instance, casino workers in Australia remain exposed to SHS in VIP areas of casinos, despite years of protests, while in the United States casinos have been excluded from some state-wide smoke-free laws [10,14]. In North America as a whole, there are debates about smoking in casinos that are part of Native American reservations, as these are not covered by state-wide legislation [10]. In England, casinos were at risk of being excluded from national smoke-free regulations, because proposals exempted private members clubs (of which casinos were part). However, following lobbying from health groups, the House of Commons voted for comprehensive smoke-free legislation covering all indoor workplaces, which came into force on July 1^st ^2007 [15].

Exposure to SHS in the workplace is linked with an increase in the risk of disease, including lung cancer and heart disease [16-19]. Work-based exposure to SHS appears to also have an acute impact on health, with studies of bar workers reporting high levels of respiratory and sensory irritation symptoms [20-24]. One study of casino workers suggested prevalence of symptoms is similar to that in bar workers, but the sample size was small as it involved only forty-four workers [9]. Additional research to investigate further the acute health effects among casino workers using a larger sample would be valuable when debating the inclusion of casinos in proposed smoke-free legislation. If the prime motivation for smoke-free legislation is the protection of workers, then a workforce experiencing ill-health associated with SHS exposure should not be excluded from legislation. This research aimed to determine the prevalence of respiratory and sensory irritation symptoms among the casino workforce in London, England; identify the factors associated with reporting of symptoms; and compare prevalence of symptoms with that in other workers.

## Methods

### Study design

In February 2005 we conducted a postal questionnaire survey of casino workers in London, England, aimed at investigating knowledge, attitudes and experiences relating to exposure to SHS in the workplace. Findings relating to knowledge and attitudes towards SHS are reported elsewhere [25]. We intended to sample workers using the four major UK casino companies. However they declined to co-operate with the research. We therefore made contact with the two trade unions that represent casino workers in England; the Transport and General Workers' Union (TGWU) and the GMB trade union. Both agreed to provide access to their members. We focused on casino workers in London because, unlike in other parts of England, London casino workers are highly unionised (1568 workers, around 50% of the total London casino workforce, were members of one of the two unions at the time of the survey). In addition, approximately one quarter of all casino workers in England are employed in London.

### Outcome and exposure measures

To compare the prevalence of ill-health among the casino workforce with that of other workers, our questionnaire used the same outcome measures as previous studies of bar and casino workers [9,20-24]. These questions, adapted from the International Union Against Tuberculosis and Lung Disease Bronchial Symptoms Questionnaire, ask workers to report whether they have suffered from a number of respiratory and sensory irritation symptoms in the previous four weeks [26]. In order to assess the factors associated with the reporting of symptoms, two dichotomous outcome variables were created; the reporting of one or more sensory irritation symptoms in the past four weeks; and the reporting of one or more respiratory irritation symptoms in the past four weeks.

To estimate the exposure of casino workers to SHS at work, workers were asked to rate the frequency (never exposed/sometimes exposed/often exposed/nearly always exposed) and intensity (nil/light/moderate/heavy) of their exposure to SHS in the workplace. These measures were then combined to create a binary exposure variable – reporting exposure to heavy levels of SHS nearly all the time at work, or not. This variable was hypothesised to be the main predictor of reporting respiratory and sensory irritation symptoms. Data on other personal characteristics was collected, including age, sex, smoking status, educational attainment, years worked in casinos, and average number of hours per week worked in the casino.

### Distribution of questionnaires

Ethics approval was obtained from the University of the West of England Ethics Committee in October 2004. We distributed questionnaires by post from the offices of the TGWU and GMB trade unions in February 2005 to all casino workers in London who were members of either union (across 25 London casinos). The questionnaires were completely anonymous, following concerns by the unions about confidentiality. In order to maximise the response rate we arranged for reminder posters to be posted on union notice boards in each casino, worked with health and safety representatives via the trade unions to encourage people to respond, and sent a second questionnaire with reminder letter to all workers in May 2005. Participants gave their consent for participation in the research by completing and returning a questionnaire. This was explained in the participant information sheet that was sent out with each questionnaire.

### Data analysis

Analysis was undertaken using SPSS 12.0.1. (SPSS, Chicago, IL). Data were cleaned, and potential duplicate responders were identified through a matching analysis using work history and demographic data. We conducted basic descriptive analysis. Analyses of the linear trend between frequency and intensity of SHS exposure and sensory and respiratory irritation symptoms were done by including variables for frequency or intensity of SHS exposure as continuous variables in logistic regression models. Univariate and multivariate analysis was then used to assess the relationship between SHS exposure and the reporting of one or more symptoms, adjusting for hours worked per week, living with a smoker, smoking status, years worked in casinos, gender, age, and level of educational attainment. Finally we compared the prevalence of irritation symptoms in casino workers with that reported in other workers using data from papers using the same questionnaire and identified following a review of the literature.

## Results

### Characteristics of respondents

Of the 1568 casino workers targeted, 559 responded to the survey after two mailings (response of 36%). One likely duplicate responder was identified and excluded from the analysis. Just over half of respondents were male (298, 54%), which compares with a 60% male union membership. Mean length of employment in casinos was 17 years, with an average of 38 hours worked per week. The majority of respondents worked on the gaming floor (470, 84%), while other areas of work included the reception (22, 4%) and restaurant areas (16, 3%). Of the respondents, 22% (125/559) were current cigarette smokers, while 39% (218/559) had never smoked. 12% (64/556) of respondents had degree level qualifications. The majority of workers were aged between 24 and 54.

### Self-reported exposure to SHS

The majority of workers graded their exposure to SHS in the highest category for both intensity and frequency. 83% (459/556) reported being nearly always exposed to SHS at work, while 74% (414/556) of respondents rated their intensity of exposure as "heavy". After combining estimates of frequency and intensity, 71% (393/556) of respondents classified themselves as being nearly always exposed to heavy levels of SHS at work. This combined variable was then used in the univariate and multivariate analyses, as described previously.

### Prevalence of sensory and respiratory irritation symptoms

91% (505/559) of casino workers reported the presence of at least one sensory irritation symptom in the previous four weeks, while 84% (462/559) reported one or more respiratory irritation symptoms during that time (Table 1). The three sensory irritation symptoms were the most commonly reported symptoms, with workers less likely to report respiratory symptoms (Table 1).

**Table 1:** Self-reported sensory and respiratory irritation symptoms in the last 4 weeks N = 559

Sensory and respiratory irritation symptoms	Yes N (%)	No N (%)	No Response N (%)
*Sensory irritation symptoms*			
Runny nose, sneezing or nose irritation	431 (77)	102 (18)	26 (5)
Eyes red or irritated	370 (66)	151 (27)	38 (7)
Sore or scratchy throat	368 (66)	163 (29)	28 (5)
*Respiratory irritation symptoms*			
Cough during rest of day or night	338 (61)	192 (34)	29 (5)
Short of breath	260 (47)	270 (48)	29 (5)
Bring up phlegm	246 (44)	287 (51)	26 (5)
Wheezing or whistling in chest	213 (38)	319 (57)	27 (5)
Cough first thing in morning	194 (35)	332 (59)	33 (6)

There are suggestive dose-response relationships in the odds of reporting sensory and respiratory irritation symptoms, by both self-reported frequency and intensity of exposure to SHS at work (Table 2). However, the findings are not conclusive.

**Table 2:** Adjusted‡ Odds Ratios (95% CI) for reporting sensory or respiratory symptoms by self-reported frequency and intensity of exposure to SHS at work n = 559

	Frequency of exposure to SHS at work				Intensity of exposure to SHS at work			
*Sensory irritation symptoms*	Never/Sometimes exposed	Often exposed	Nearly always exposed	Linear trend test p-value	Nil/Light	Moderate	Heavy	Linear trend test p-value
Red or irritated eyes	1.00	1.22 (0.43 to 3.48)	4.01 (1.63 to 9.88)	< 0.0001	1.00	1.49 (0.57 to 3.90)	5.95 (2.36 to 14.96)	< 0.0001
Runny nose, sneezing or nose irritation	1.00	1.49 (0.53 to 4.20)	3.10 (1.28 to 7.50)	0.008	1.00	2.54 (0.98 to 6.60)	4.02 (1.63 to 9.94)	0.006
Sore or scratchy throat	1.00	0.45 (0.16 to 1.23)	1.49 (0.62 to 3.59)	< 0.0001	1.00	1.24 (0.50 to 3.07)	3.25 (1.37 to 7.74)	< 0.0001

*Respiratory irritation symptoms*								
Wheezing or whistling in chest	1.00	1.64 (0.57 to 4.71)	1.99 (0.80 to 4.94)	0.30	1.00	1.45 (0.52 to 4.01)	2.47 (0.94 to 6.46)	0.03
Felt short of breath	1.00	0.49 (0.18 to 1.33)	1.19 (0.52 to 2.71)	0.02	1.00	1.29 (0.49 to 3.37)	2.88 (1.16 to 7.13)	0.001
Cough first thing in morning	1.00	1.14 (0.40 to 3.25)	1.48 (0.60 to 3.63)	0.53	1.00	2.86 (0.88 to 9.36)	4.14 (1.33 to 12.94)	0.02

‡ Odds Ratios adjusted for smoking status, living with a smoker, years worked in casinos, gender, age, and highest level of qualifications using multivariate logistic regression analysis.

In multivariate analysis, after controlling for other variables in the model, the most important determinant of reporting sensory or respiratory irritation symptoms was exposure to SHS at work (Table 3 and 4). Non-smokers were also more likely than smokers to report sensory irritation symptoms, as were those with degree or higher educational qualifications compared to those with the lowest qualifications (Table 3). In addition to exposure, the presence of respiratory symptoms was associated with the number of hours worked per week (Table 4).

**Table 3:** Odds Ratios (OR) for reporting the presence of at least one sensory irritation symptom over the past four weeks, from univariate and multivariate analyses

	N	Unadjusted OR (95% CI)	Adjusted OR (95% CI)‡
**Hours worked per week**		0.99 (0.96 to 1.04)	1.01 (0.96 to 1.05)
**Exposure to SHS at work:**			
Not heavily exposed nearly all time	155	1.00	1.00
Heavily exposed nearly all the time	376	4.19 (2.33 to 7.55)	3.26 (1.72 to 6.16)
**Household smoking:**			
Does not live with smoker	412	1.00	1.00
Lives with smoker	119	1.68 (0.89 to 3.15)	1.08 (0.48 to 2.46)
**Smoking status:**			
Smoker	120	1.00	1.00
Non-smoker	411	2.31 (1.27 to 4.19)	2.70 (1.21 to 5.99)
**Years worked in casinos**		0.98 (0.95 to 1.01)	0.99 (0.93 to 1.03)
**Gender:**			
Male	280	1.00	1.00
Female	251	1.77 (0.98 to 3.20)	1.52 (0.77 to 2.97)
**Age:**			
16–34	148	1.00	1.00
35–54	313	0.51 (0.24 to 1.08)	0.70 (0.26 to 1.90)
55+	70	0.50 (0.17 to 1.18)	1.41 (0.31 to 6.34)
**Highest qualifications†:**			
GCSE D-G or lower	103	1.00	1.00
Degree or higher education qualif	127	3.87 (1.47 to 10.21)	3.09 (1.10 to 8.73)
A levels or ONC/BTEC	144	2.13 (0.97 to 4.66)	2.06 (0.85 to 4.97)
GCSE A-C or equivalent	157	1.69 (0.81 to 3.51)	1.43 (0.63 to 3.22)

‡All Adjusted Odds Ratios adjusted for other variables presented in the table, using multivariate logistic regression analysis.

†GCSE are qualifications usually taken at the age of 16, at high school. A-levels and ONC/BTEC qualifications are usually taken at the age of 18.

**Table 4:** Odds Ratios (OR) for reporting the presence of at least one respiratory irritation symptom over the past four weeks, from univariate and multivariate analyses

	No	Unadjusted OR (95% CI)	Adjusted OR (95% CI)‡
**Hours worked per week**		1.03 (1.00 to 1.07)	1.04 (1.00 to 1.07)
**Exposure to SHS at work:**			
Not heavily exposed nearly all time	151	1.00	1.00
Heavily exposed nearly all the time	372	2.41 (1.50 to 3.86)	2.24 (1.34 to 3.74)
**Household smoking:**			
Does not live with smoker	404	1.00	1.00
Lives with smoker	119	1.50 (0.82 to 2.71)	1.43 (0.70 to 2.93)
**Smoking status:**			
Smoker	119	1.00	1.00
Non-smoker	404	0.68 (0.37 to 1.23)	0.74 (0.36 to 1.53)
**Years worked in casinos**		1.00 (0.97 to 1.02)	1.01 (0.97 to 1.05)
**Gender:**			
Male	275	1.00	1.00
Female	248	1.08 (0.68 to 1.72)	1.01 (0.60 to 1.68)
**Age:**			
16–34	148	1.00	1.00
35–54	306	0.81 (0.47 to 1.40)	0.92 (0.44 to 1.92)
55+	69	0.61 (0.30 to 1.27)	0.86 (0.30 to 2.50)
**Highest qualifications†:**			
GCSE D-G or lower	102	1.00	1.00
Degree or higher education qualif	125	1.51 (0.77 to 2.97)	1.39 (0.67 to 2.90)
A levels or ONC/BTEC	143	1.49 (0.78 to 2.87)	1.30 (0.64 to 2.64)
GCSE A-C or equivalent	153	1.69 (0.88 to 3.27)	1.48 (0.73 to 3.01)

‡All Adjusted Odds Ratios adjusted for other variables presented in the table, using multivariate logistic regression analysis.

†GCSE are qualifications usually taken at the age of 16, at high school. A-levels and ONC/BTEC qualifications are usually taken at the age of 18.

The prevalence of respiratory and sensory irritation symptoms among the casino workers in this sample was higher than that found in the casino worker study in Australia (Table 5) [9]. The prevalence of symptoms in casino workers also tended to exceed that in bar workers documented in previous studies, whether non-smokers (as with the bar workers in New York and Ireland), or all workers (as in California) (Table 5) [20-23]. Prevalence was similar to that found in bar workers in Wisconsin [23].

**Table 5:** Prevalence (%) of self-reported sensory and respiratory irritation symptoms, comparison of workforce studies

Sensory and respiratory irritation symptoms	London casino workers (all) n = 559	London casino workers (non smokers) n = 434	London casino workers (smokers) n = 125	Palmersheim *et al*, 2006, Wisconsin bar and restaurant workers** n = 230	Allwright *et al*, 2005, Ireland bar workers** n = 138	Eisner *et al*, 1998, California bar workers* n = 53	Wakefield *et al*, 2005 Victoria casino workers** n = 44	Farrelly *et al*, 2005, New York bar workers** n = 24
*Sensory irritation symptoms*								

Eyes red or irritated	66	70	52	70	41	42	66	67
Runny nose, sneezing or nose irritation	77	79	72	78	44	60	61	54
Sore or scratchy throat	66	69	54	61	33	25	52	42

*Respiratory irritation symptoms*								

Wheezing or whistling in the chest	38	38	38	31	21	32	20	21
Short of breath	47	47	44	41	16	19	23	17
Usually cough first thing in the morning	35	31	48	43	21	53	23	21
Cough at all during the rest of the day or night	61	59	64	52	38	49	30	29
Bring up any phlegm	44	43	49	53	43	53	25	21

* 45% of the workers were current smokers but results were only presented jointly

** workers were non-tobacco users

Studies were pre-ban in Ireland and New York

## Discussion

### Main findings of this study

This study has found that the majority of casino workers in London who participated in the survey reported at least one sensory and respiratory irritation symptom in the previous four weeks. Reporting the presence of irritation symptoms was most strongly associated with reporting the highest level of exposure to SHS at work. The apparent link between exposure to SHS and respiratory and sensory irritation symptoms is strengthened by the suggestive dose-response relationship between self-reported frequency and intensity of exposure and both symptom types.

The association between high levels of sensory and respiratory irritation symptoms and exposure to SHS is consistent with that found in other studies of workers. Evidence is particularly strong in the studies of bar workers that demonstrated decreases in prevalence of symptoms after the introduction of smoke-free policies. Most decreases were statistically significant compared to baseline [20-24]. As reported elsewhere, many casino workers in this study believed that SHS affected their health, with 57% (315/558) believing that their health had been affected by exposure to SHS at work [25].

Our research demonstrates that among this sample of casino workers, the prevalence of respiratory and sensory irritation symptoms is generally higher than those reported in studies of bar workers and the previous study of casino workers. This could be because of differing exposure to SHS between casino and bar environments. As the studies of bar workers suggest that workers report similar frequency of exposure to SHS whilst in the workplace, the higher prevalence may relate to longer working hours and greater intensity of exposure in casino workers. Casino workers are in their workplace for a greater proportion of the working week, working an average 38 hours per week compared to a range of 21–40 hours per week for bar workers [20-23]. Unlike pubs or bars, casinos often do not have windows or other sources of fresh air and our previous research has revealed that casino workers are often face to face with several smokers at gaming tables for significant periods of time [25]. The difference in prevalence of symptoms between our study and the previous research on casino workers may be because of the smaller sample size in that study and/or the fact that 5% of the casino workers reported that smoking was banned at their workstation (suggesting that this workforce may have been exposed to less SHS than the London sample) [9].

As noted, our study also found associations between symptoms and other variables. The association between working for more hours per week in the casino and respiratory irritation symptoms is logical, as this could be viewed as an alternative measure of exposure; suggesting a higher frequency of exposure to SHS during the working day. However there was little variation in average hours worked in casinos between workers, which may explain why average hours worked was only weakly associated with respiratory symptoms and not associated with sensory irritation symptoms. The association between smoking status and sensory irritation symptoms (but not respiratory symptoms) may be because sensory symptoms are primarily associated with SHS (of which smokers might be less affected), while respiratory symptoms are related more closely to the effects of active smoking. The study of bar workers in Wisconsin also found this association between smoking status and symptoms [23]. It is not clear why there is a link between having degree or higher educational qualifications and reporting sensory irritation symptoms.

### What this study adds

To our knowledge this is the first study to estimate prevalence of sensory and respiratory irritation symptoms among a large sample of casino workers. As most studies of respiratory and sensory irritation symptoms have focussed on bar workers, it offers a valuable insight into how exposure to SHS affects other exposed workforces. This is particularly important when those workforces are at risk of being excluded from smoke-free legislation. The sample size of 559 workers is larger than that of previous studies of bar and casino workers. As such, the paper provides further evidence using a larger population that exposure to SHS in the workplace is associated with reporting of both sensory and respiratory irritation symptoms.

The lack of other such studies might be in part due to the difficulties in accessing casino workers. We have already outlined how the casino companies refused to co-operate in our research, and it is likely that other such employers around the world may be reluctant to allow access to employees for fear of bad publicity and worker unrest. Indeed, without the option of sampling through trade unions, this workforce would not have been accessible to us. It may be that in other parts of the world, trade unions offer a route to access otherwise hard to reach working groups such as casino workers. In the debates on smoke-free legislation around the world, it is vital that the voices of those workers who are exposed to high levels of SHS at work are heard.

### Limitations of this study

This study has several limitations. The main limitation relates to the potential of responder bias among the sample of casino workers. As outlined, the survey had a response of 36%. Unfortunately, other than gender, the unions held no other summary data on their members against which to assess the representativeness of the responders to the targeted unionised population. It was also not possible to assess how responders compared to the wider casino workforce in London, due to being unable to obtain such data from the casino companies.

Although the proportion of smokers among the respondents (22%) is comparable to national UK estimates of smoking prevalence, there is likely to be under-representation of smokers in the study results [27]. A previous study in London estimated that 37% of manual workers were current smokers, and casino workers are likely to fall in this group [28]. An underestimation of smokers among the respondents would affect overall estimates of the prevalence of irritation symptoms, as smoking status is an important variable in the logistic regression. However, it is not possible to determine whether there is under-representation, as data on smoking prevalence among casino workers in the United Kingdom is not collected either nationally or by the trade unions.

It is possible that those who were suffering from ill-health that they associated with exposure to SHS were more likely to respond to the survey. In this scenario, the prevalence of respiratory and sensory irritation symptoms among casino workers may be overestimated. Unfortunately it is not possible to determine if this occurred. Other studies of workers also note weaknesses relating to possible responder bias [20-23]. These studies, like ours, lacked the necessary baseline data to assess whether such bias was likely.

Our study relied on self-reports of exposure to SHS. This measures perception of exposure to SHS, not actual exposure. Existing evidence suggests that while workers and others are usually able to report accurately on whether they are exposed to SHS or not, quantifying the extent of their exposure is more problematic [29]. In order to validate the self-reported measures of exposure, an objective measure of exposure to SHS could have been gathered from each worker, such as a before- and after-shift cotinine sample. However, resource limitations meant that this was not feasible.

Our study and previous studies have used respiratory and sensory irritation symptoms as indicators of acute health effects of SHS. Symptoms such as coughing and having a sore throat and runny nose could also be associated with other causes, such as bacterial and viral infections. We did not control for the presence of cold or other respiratory conditions. However, the previous study of casino workers did find an association between exposure to SHS and symptoms, after adjusting for presence of cold and other respiratory conditions [9]. In addition, the observed decreases in respiratory and sensory irritation symptoms in the bar worker studies following the introduction of smoke-free legislation (and accompanying decreases in cotinine levels) strengthens the likelihood that SHS exposure does play a large role in the prevalence of these symptoms.

### Implications of the findings

The existing evidence that SHS is harmful to health is already sufficient to demonstrate the need for smoke-free legislation that protects all workers, including those in casinos. In fact, the recent legislation in England now means that the casino workers surveyed as part of this research are now protected from SHS at work, as are those workers in countries such as Ireland, Italy, and Norway [11-13,15]. Unfortunately, the decision to exclude casino workers from smoke-free legislation in some parts of the United States and the creation of exempted areas in casinos in Australia demonstrates that some policy makers continue to ignore the health needs of the most vulnerable workers.

The findings reported here emphasise that while casino workers represent a working group that is most likely to be excluded from smoke-free legislation, they report high levels of ill-health associated with exposure to SHS. Unless smoke-free legislation is comprehensive, it will fail in its aim to protect workers and improve public health; instead, widening health inequalities by leaving the most vulnerable workers at risk. Inequalities will not only widen because of the direct health effects of exposure to SHS, as it is known that when smoke-free policies are introduced in workplaces, smokers are more likely to cut down or quit completely [30]. Those smokers working in settings that are excluded from legislation will be denied this benefit.

## Conclusion

In this study casino workers who were exposed to SHS at work reported high levels of sensory and respiratory irritation symptoms. The prevalence of symptoms is in general higher than that reported in previous studies of workers. Our study also demonstrates an association between self-reported exposure to SHS and reported irritation symptoms, including a suggestive dose-response relationship. Given the before-after reductions in respiratory and sensory irritation symptoms observed in bar workers following introduction of smoke-free legislation, the potential health gains for casino workers are significant. Our research supports the need for comprehensive smoke-free legislation worldwide, covering all indoor workplaces. Those workers who are most exposed to SHS at work, such as casino workers, are likely to suffer the greatest from its health effects and benefit most from future smoke-free policies.

## Competing interests

The author(s) declare that they have no competing interests.

## Authors' contributions

PP designed and conducted the questionnaire survey, with ongoing advice from SG and AG. PP drafted the first version of the paper. PP revised the paper following comments and edits from SG and AG. All authors read and approved the final draft of the paper.

## Pre-publication history

The pre-publication history for this paper can be accessed here:


